# Towards Establishing Criteria for the Ethical Analysis of Artificial Intelligence

**DOI:** 10.1007/s11948-020-00238-w

**Published:** 2020-07-07

**Authors:** Michele Farisco, Kathinka Evers, Arleen Salles

**Affiliations:** 1 Centre for Research Ethics and Bioethics, Uppsala University, Box 564, 751 22 Uppsala, Sweden; 2 Science and Society Unit, Biogem, Biology and Molecular Genetics Institute, Via Camporeale, Ariano Irpino, AV Italy; 3 Programa de Neuroetica, Centro de Investigaciones Filosoficas, Buenos Aires, Argentina

**Keywords:** Ethics of artificial intelligence, Ethics of robotics, Philosophy of artificial intelligence, Philosophy of intelligence, Biological intelligence

## Abstract

Ethical reflection on Artificial Intelligence (AI) has become a priority. In this article, we propose a methodological model for a comprehensive ethical analysis of some uses of AI, notably as a replacement of human actors in specific activities. We emphasize the need for conceptual clarification of relevant key terms (e.g., intelligence) in order to undertake such reflection. Against that background, we distinguish two levels of ethical analysis, one practical and one theoretical. Focusing on the state of AI at present, we suggest that regardless of the presence of intelligence, the lack of morally relevant features calls for caution when considering the role of AI in some specific human activities.

## Introduction

AI is a rapidly expanding area of development and application. Aware that there is no general agreement on how to understand it,^1^ we follow here the definition provided by a recent Communication by the European Commission, according to which AI “refers to systems that display intelligent behavior by analyzing their environment and taking actions—with some degree of autonomy—to achieve specific goals” (European-Commission 2018). Because this definition is formal and general enough to cover most common understandings of the field, it constitutes a useful starting point. Indeed, it is on the basis of this understanding that the High-Level Experts Group of the European Commission developed a more granular definition of AI as “systems designed by humans that, given a complex goal, act in the physical or digital world by perceiving their environment, interpreting the collected structured or unstructured data, reasoning on the knowledge derived from this data and deciding the best action(s) to take (according to pre-defined parameters) to achieve the given goal. AI systems can also be designed to learn to adapt their behavior by analyzing how the environment is affected by their previous actions” (AIHLEG 2018).

Beyond the existing controversy concerning the definition of AI, it is possible to identify the following elements as crucial to its functioning: perception of the environment through sensors; reasoning/decision making on data perceived; and actuation through executors.

AI so conceived opens several possibilities in different fields, from medicine to the military, raising multiple ethical issues. To illustrate shortly, advances in AI and automatization might enable more pervasive monitoring, surveillance, and tracking of people and their activities, as well as the faster distribution of information throughout the world, which raises several security and privacy challenges (Stahl and Wright 2018). Furthermore, the potential facilitating role of AI in medicine, in the military, and in the development of autonomous weapons gives rise to issues about unreliability and accountability (Hammond 2015; Hallaq et al. 2017; Horowitz 2018). Moreover, together with automatization, AI is likely to bring forth far-reaching economic and social changes, making an impact on the labor market in general (Aicardi et al. 2018). What kinds of jobs are likely to be affected is still debated; however, there is agreement that AI will transform work demands and the nature of those positions that depend on human abilities and skills (Perisic 2018). It may do so by possibly creating new ones and eliminating extant others (EGE 2018), and making a significant impact on traditional hiring and recruitment practices through, for example, predictive analytics.

Pressing ethical issues also emerge in one of the most advanced types of AI research: self-learning AI. Self-learning AI learns from reinforcement without human data, guidance, or domain knowledge beyond basic rules (Silver et al. 2017). According to its developers, without any previous specific knowledge self-learning AI can achieve “superhuman proficiency in challenging domains” (Silver et al. 2017). So far, this kind of AI has been successfully applied within limited contexts, notably in games like Chess, Go, and Poker, where all the relevant variables can be predicted, even if through extremely sophisticated calculations. The application of these systems in real-world environments (e.g., self-driving cars) raises challenging problems that have not been solved yet, although we cannot rule out the possibility that their solution is just a matter of time (Sokol 2018).

Without speculating on “big ethics” concerns that focus on large scale potential scenarios (e.g., superintelligent AI taking over) (Muller 2016), it is clear that AI is increasingly socially relevant through its many current uses and its capacity to perform (in some contexts) better than humans. This is beginning to cause some psychological uneasiness and might contribute to a shift in some of the culturally shaped conceptual frameworks people use to think about themselves as human and ethical beings, about machines’ ontological and moral status, and the human–machine relationship (Tegmark 2018). While the ontological and moral distinction between human beings and the objects they build and use has typically been taken for granted, some recent technological advances such as Brain Machines Interfaces seem to challenge it (Schermer 2009). Similarly, advances in artificial intelligence are likely to affect not just the familiar conceptual framework that tends to make a sharp distinction between the natural and the artificial, but also the generally accepted biocentric view that only living organisms can be moral subjects (Attfield 2016).

Before addressing these issues, it is useful to clarify what we understand ethical reflection to encompass. The umbrella of ethics includes normative ethical analysis (i.e., oriented to the assessment of practical issues concerning, for instance, the social impact of science) but notably it also includes reflection on more theoretical issues (i.e., a conceptual analysis of key notions to assess, for instance, the relevance of science to philosophical, social, and ethical concerns). Thus, in our view, to better address the practical issues potentially arising from AI, it is important to examine how the discussion is framed and to clarify the most relevant concepts. One of the core concepts in need of attention is, of course, “intelligence,” as are the distinction between natural and artificial intelligence, and the connection between intelligence and consciousness. Understanding the meaning and significance of intelligence in this context is a prerequisite for correctly identifying and assessing many relevant and often pressing ethical and social issues directly connected to the emergence of AI. Therefore, in this article, we begin with an analysis of this notion. Since this is a work in progress, some issues, notably the relationship between intelligence and consciousness and between intelligence and emotions, even if mentioned when relevant to the topic discussed, will not be directly addressed. Moreover, because of space constraints, we do not attempt an exhaustive examination of the concept of intelligence. We begin by focusing instead on some aspects that are particularly relevant for an ethical reflection on AI.

Next, we offer a methodological model for an ethical analysis of some particular uses of AI. We argue that such analysis should be carried out at two levels: an applied socio-practical level that focuses on, for example, the impact of AI on healthcare, education, and the job market, and a more theoretical level that includes fundamental ethical questions about the moral significance and ethical relevance of AI. Thus, our main concern is not with providing a specific answer to applied issues raised by AI but rather with emphasizing that a preliminary conceptual clarification of intelligence is relevant if not critical for properly assessing some practical and fundamental ethical issues raised by the field and its applications.

## Conceptual Analysis of Intelligence

There is no agreement on what intelligence is and what it entails (for an overview see (Legg and Hutter 2007)). This is probably the reason why some AI researchers, including Alan Turing, either do not explicitly define intelligence in their work, preferring to focus instead on how intelligent action is manifested (Prescott and Camilleri 2018), or provide a working definition of intelligence, sufficient for their purposes but possibly insufficient for an ethical analysis of AI and its social and economic impacts.

Furthermore, there is a tendency to consider that AI systems that are manifestly superior to human agents in the performance of some specific task (e.g., calculus, memory, strategy tasks like playing chess or Go, etc.) are not "really" intelligent. This might be related to several factors, for example, a general reluctance to apply what is arguably a mental term to machines, coupled with a fear of the unfamiliar, or some residual anthropocentrism or biocentrism (more on this point in the ethics discussion below). Underlying the idea that some specific abilities are not really intelligent is the distinction between general and narrow intelligence, i.e., between the capacity to solve a whole array of problems in different domains and the capacity to do so in a narrowly defined domain. This differentiation has been widely recognized and accepted within AI communities, as illustrated by the widespread use of the distinction between artificial general intelligence (AGI) (Goertzel and Pennachin 2007; Goertzel and Wang 2007)—strong or full AI—and narrow—classical, or weak—AI (Kurzweil 2005).

From a conceptual point of view, two main approaches to defining intelligence prevail. One defines intelligence contextually: intelligence depends at least partly on the interaction between subject and surroundings. The other understands intelligence as intrinsic to the subject independent from any context or interaction. While traditionally an intrinsic understanding of intelligence has prevailed (for example, in Greek Philosophy, particularly from Anaxagoras to Plato, and then from Neo-Platonism to Idealism—both classical and contemporary—(Adams 2007; Hösle 1998)), the contextual approach is gaining traction in contemporary discussions (Goertzel and Wang 2007).

### Intelligence in the Life Sciences

Within the life sciences, intelligence has been described as the ability of an organism to solve problems emerging in its natural and social environment (Roth 2013). Gerhard Roth defines intelligence as behavioral flexibility and innovation capacity. Both abilities subserve the final aim of the intelligent behavior of a living organism: self-preservation. A biological organism has an intrinsic ability to interact with its surroundings in order to preserve itself, and its intelligent behavior can be instantiated in different specific features, e.g., associative learning, memory formation, behavioral flexibility, innovation rate and abilities requiring abstract thinking, concept formation, and insight. These abilities do not necessarily require awareness: some can also be found in very simple organisms, like unicellular organisms that possess the capacity for learning, memory, and other multisensory information processing (Roth 2013). Those features underlying the behavior of very simple organisms have been labeled “minimal intelligence” (Calvo and Baluška 2015). Still, even if minimal, in this context intelligence is a skill for surviving through behavioral flexibility and innovation capacity.

What drives biological intelligence, as described above, is the need that the biological organism perceives and strives to satisfy. Such perception might be either aware or unaware since the experience of a need to be satisfied is independent of actual awareness by the subject. This suggests the possibility of intelligence without awareness, where intelligence is understood as the ability to achieve goals through behavioral flexibility and innovation capacity but not necessarily with the reflexivity required by the awareness of one’s own experiences.

Within the biological realm, emotions connect intelligence to goals (LeDoux 1998; Evers 2009a). While the topic of what emotions are is still widely debated, for the sake of simplicity here we can refer to emotions as the brain’s capacity to attach value to internal representations or to external inputs where values are understood as responses to reward signals, factors that affect and determine choices, selections, and decisions (Evers 2009a). In short, emotions indicate the different degrees of relevance and adequacy of both internal and external inputs. The evaluation and “anticipation of reward” introducing a delay between the elaboration of tacit plans of action and the actual interaction with the world performed by the organism is an important stimulus of learning (Changeux 2004).

### Intelligence in AI Research

The connection of intelligence to goals is generally acknowledged and referred to by AI researchers: goals achievement, adaptation to environmental conditions, and behavior flexibility are among the features AI developers attribute to their products when labeling them intelligent (Legg and Hutter 2007; Pennachin and Goertzel 2007; Wang 2007).

Within AI, initially, intelligence was identified with specific tasks and with the relevant internal processes considered necessary for humans to perform those tasks (Russell 2016). The concept was developed within a functionalist framework: the main idea was that cognition is a computational process over symbolic representations (i.e., computationalism) (Brooks 1990). The main goal of AI research was to learn more about natural intelligence by discovering and replicating these computations artificially. Accordingly, AI systems were conceived as deterministically programmed to instantiate specific actions. However, starting in the 1990s, AI research goals changed: the aim became building intelligent agents, i.e., “entities that sense their environment and act upon it” (Russell 2016). Within this new framework, AI is not necessarily or completely connected to algorithms (i.e., top-down instructions) for decision-making, but rather an adaptive process of computation interacting with the environment for more efficient decision-making, that is, a kind of bottom-up decision process implemented through reward signals (Russell 2016).

Accordingly, at present intelligence in AI is not considered simply a computational process of symbols according to specific instructions, but rather an adaptive and flexible interaction with the environment.

### Similarities and Differences Between Intelligence in the Life Sciences and AI Research

Notwithstanding the move from a naively computational/functionalist concept of intelligence to an interactive and contextual view, differences between the definition of intelligence within the biological sciences and within AI research still exist. It is true that behavioral flexibility and innovation capacity, which a biological account of intelligence recognizes as critical, might also be expressed, at least partly, by AI. However, the needs and goals identified as constitutive of these abilities are, to date, substantially different in biological organisms and in AI. In the first case, needs and goals originate from the capacity of attaching values and having preferences among external stimuli, i.e., from an emotional interaction with the environment. Admittedly, AI may be able to recognize/label human emotions, but it is doubtful that AI understands them in the sense of feeling with others, and that it uses this capacity, for example, in situations of care or education. To use emotions in those contexts presupposes a form of emotional and social intelligence (Gardner 1985; Goleman et al. 2004) as well as a theory of mind. These abilities are often critical for making the most appropriate choices to actual situations even when they are not necessarily consistent with strict logical reasoning, or in some cases are even counterintuitive.

## Ethics of AI

The ethical reflection on AI is growing rapidly (EGE 2018; Floridi et al. 2018; Bentley et al. 2018), but it seems mostly focused on practical and normative issues. As previously stated, while recognizing the importance of addressing applied issues, an examination of key concepts and reflection on more fundamental ethical questions is paramount (Farisco et al. 2018).

Why and how is the conceptual examination of natural and artificial intelligence ethically and socially relevant? Starting with concrete, practical issues, there are at least two important areas where the distinction between natural and artificial intelligence might have an impact: healthcare and education (i.e., a child-care facilities setting). We focus on these two areas for illustrative reasons: while they do not exhaust all the potential issues raised by AI, they do allow us to point to features potentially relevant in other contexts as well, particularly those where AI might be used to replace human actors.

The conceptual analysis above is also relevant to addressing more theoretical concerns such as whether and how AI might affect traditional ethical notions related to human intelligence to which human beings attribute special value. To illustrate, how would the existence of self-learning and possibly autonomous AI change the way humans think about their own supposedly unique status?

We address both practical and more theoretical concerns next.

### Practical Ethical Concerns

Practical ethical issues emerging from AI generally revolve around the impact that AI has on different sectors of society. Specific practical concerns include issues such as the potential replacement of humans by AI in the labor market, hype concerning AI abilities, humans loss of some skills due to overreliance on AI for a growing number of activities, increasing changes in human interactions, and AI’s potentially addictive design, among others. The analysis of intelligence provided above is relevant to a practical ethical discussion of this topic because it provides cues that allow us not only to reflect on what actions humans tend to value but also to assess some concerns raised by AI. We would like to propose that if there are some features typically considered critical for carrying out actions in an ethically sound way in certain contexts (e.g., assisting elderly patients) and AI lacks those features, then using AI to replace humans in those contexts might be questionable.

*Prima facie*, there are two important aspects regarding human and artificial intelligence that merit attention. First, as suggested above, while the human brain is emotional in the sense of having preferences that determine its operations (LeDoux 2003; Evers 2009b) and is *ipso facto* importantly organized by emotional responses such as sensitivity to reward signals, AI is not (yet) emotional in this manner. This is not just relevant from an ontological perspective. From a moral perspective, and *contra* a historically prevalent view that tends to reduce the moral value of an action to its rationality, several authors have argued not only that rationality is never clean of emotional influence, but also that such emotional influence can play a positive role when assessing the ethical quality of actions. Even further, some argue that an action motivated and accompanied by the appropriate emotional involvement might actually be ethically superior to one that lacks such motivation (Oakley 1992; Stocker and Hegeman 1996; Blum 1980; Blackburn 1998). This point has been consistently made in a number of contexts, including the medical context. In fact, some commentators hold that assisting a patient without any empathy or emotional involvement is morally different from doing so empathetically. The reason for this is not necessarily because the possession of some emotions might be intrinsically desirable but rather because emotional involvement directs our attention to certain issues that should be looked into. Empathy often functions as a moral compass and source of insight providing unique access to others’ needs (Nussbaum 2001). It is true that in some specific contexts, such as healthcare and education, the most effective actions in terms of the most beneficial effects on recipients are those where caregivers or educational staff show the most emotional empathy (Neumann et al. 2009, 2012; Halpern 2001). If this is the case, then AI’s lack of emotional involvement should be taken into account when considering whether AI can replace humans in activities that require assisting vulnerable persons in the healthcare, child-care, and senior care settings.

Second, while compelling, the view of intelligence as an interactive process with the environment that underlies the most advanced AI risks reducing intelligence simply to a reaction to environmental inputs. Such interpretation is challenged by pictures of the brain emerging from contemporary neuroscience according to which the brain is not simply reactive but spontaneously, intrinsically active, and able to develop its models of the surrounding world that anticipate actual experiences (Friston 2010; Changeux 2004; Farisco et al. 2017).

This is significant notably because the intrinsic activity of the brain enables a salient human capacity: the capacity for abductive reasoning, i.e., of counterintuitive inferences. That the human brain is not just an input–output machine allows for an implicit understanding of how things work that, while hard to demarcate, is highly relevant in many situations, i.e., to inter-personal human behavior.

Spontaneous modeling activity and the ability to go beyond actual experience through abstract thinking imply in particular that human (and possibly natural) intelligence is not only inductive but also abductive. This means that human (and possibly natural) intelligence does not only interpret incoming stimuli solely based on actual experience but does so within a broader framework which occasionally might justify counterintuitive conclusions. To give an example, when a human is crying, the spontaneous inference is that he is in pain, but several other possibilities that may be less or even counter-intuitive might explain the behavior better (e.g., it might be a cry of joy, or the cry might simply be the only way to express a specific need/desire, e.g. in the case of babies, etc.).

Indeed, sometimes the most intelligent (i.e., the most appropriate given the context) action is the less logically evident action but the one suggested by counterintuitive inferences aided by different capabilities. To date, this type of inferential thinking is out of reach for AI (Rogan 2019). Importantly, this fact might establish an ethically relevant difference between natural and artificial intelligence. Notwithstanding several attempts in the history of AI since the origin of the field (Thòrisson and Kremelberg 2017), the inability to make counter-intuitive inferences on the basis of what is usually understood as “common sense” (implicit widely shared knowledge that allows us to predict and explain human behavior) might expose AI to the risk of “artificial stupidity”, i.e. making decisions that are evidently inappropriate to the actual situation.

These two presently existing differences between human (and possibly natural) intelligence and AI (the capacity for emotional involvement and the capacity to make counter-intuitive or inferential decision) are ethically relevant in practical terms, particularly in healthcare contexts where the physiological parameters that AI usually monitors are not sufficient to reveal the actual decision-making process and, ultimately, decisions of patients (e.g., a patient might be afraid to receive a medication even though he rationally knows it is good for his health and wants it). Alternatively, consider the involvement of AI in senior (Foster 2018) or childcare facilities (Weller 2017; Demetriou 2017), where emotional relationships are among the most important elements for adequate assistance or education.^2^ Seniors often suffer from progressive loss or impairment of their psychological identities, e.g., their most significant memories. At least to date, the relief coming from the emotional empathy humans can express cannot be replaced by AI-based systems, like robots. One can speculate about the possibility of instructing robots to simulate positive emotions by giving them all the relevant personal details necessary for the patient´s comfort, but, at least to date, they would still lack critical emotional involvement. This emotional lack is also relevant when considering the use of robots in education, notably in childcare. First, their lack of emotional involvement might result in inappropriate decisions, such as misreading children's facial expressions (like the crying referred to above) and eventually misidentifying their needs. Second, as unemotional caregivers, AIs might deprive children the possibility to experience some emotions at a critical developmental age.

Of course, it could be argued that AI-based systems like robots are increasingly equipped to express a wide range of simulated emotions (Hall 2017) and that insofar as seniors and children recognize the expression of simulated emotions in robots, they would not be deprived of the required emotional experience when interacting with them. Yet, the fact that at least at present robots are only able to simulate emotions and not experience them raises additional ethical issues such as the ethical permissibility of deception (both detected and undetected). The long-term consequences of such systematic deception have not been examined, and this is a topic that calls for some critical reflection.

The considerations above are not intended to suggest that AI should be excluded from particular societal domains such as healthcare or education. Instead, they call for caution. At least to date, AI has been unable to fully replace humans in particularly sensitive activities, even if AI can assist humans in such activities: the multidimensionality of human intelligence, including also an emotional form of intelligence, is so far not fully replaceable artificially. Whether this limitation of AI is intrinsic to the logic and structure of AI itself or rather a question of further development of the relevant technology remains a topic of debate.

The above-sketched conceptual distinctions thus suggest a possible criterion for a practical ethical analysis of some uses of AI, namely regardless of the presence of intelligence, the lack of features that humans consider to be ethically valuable needs to be addressed before considering replacing humans with AIs in some specific activities.

### Theoretical Ethical Issues

In addition to the above, there are ethical issues raised by the interactions between humans and AI that require more theoretical analyses of fundamental ethical beliefs. One such issue is whether core ethical concepts typically reserved for biological organisms can justifiably be attributed to AI as well. Specifically, can notions deemed critical in attributing moral significance and ethical relevance to biological entities also be applied to AI? Intelligence very broadly understood, including emotional and social intelligence, is one such notion. There is widespread agreement in that, minimally, the ability to choose and behave autonomously is critical for being a moral agent (i.e., having full moral agency) while having interests is critical for ethical relevance (i.e., deserving moral consideration). If this is true, insofar as to date AI does not seem able to either express autonomy or have interests, it is reasonable to conclude that it is not (yet) a moral agent nor is it ethically relevant, unless we use additional considerations in support of a different conclusion.^3^ Some commentators have argued that the possibility of AI becoming increasingly autonomous means that it should receive moral instruction, that is, be taught to distinguish between right and wrong, and act accordingly (Wallach and Allen 2009). However, the proposal that we build morally competent AI raises at least two concerns. In the first place, even if possible, it is doubtful that moral instruction would result in AI (as presently available) becoming “moral agents” if by this we mean having the capacity to give reasons and explanations for one’s decisions and actions (Boddington 2017). The process of justifying a moral choice is different from the top-down process of applying algorithmic rules to predefined notions: moral justification involves abductive, counterintuitive, and emotional steps, which seem beyond the ability of actual AI. Theoretically possible, but still controversial, is the hypothesis that AI will acquire a peculiar capacity of moral reasoning different from humans’ moral capacity. Although it cannot be theoretically ruled out, from a technical standpoint, the probability of AI acquiring moral and ethical capacities seems low at present.

In the second place, talk about the technical likelihood or unlikelihood of morally competent AI does not solve other issues such as whether there are good reasons for endowing AI with moral capacities and whether such morally competent AI is actually needed and desirable (van Wynsberghe and Robbins 2019).

Another important ethical concern raised by the interaction between humans and AI does not revolve around the ethical status of AI but rather around the issue of how AI may affect humans’ self-perception as ontologically and morally unique. The increasing use of AI in a variety of contexts, including those where human qualities have traditionally been taken to be necessary, may affect the way we perceive ourselves and even blur the ethical line between humans and AI. If, for instance, as more “intelligent” activities that are usually considered to be human-specific are carried out by AI systems, humans might question what being human is, what it means, and in what sense humans are unique. These questions become more pressing with the advancement of some subfields of AI research, notably self-learning AI. Systems based on machine learning methods, such as self-learning AI, make it possible to attribute to AI abilities usually considered uniquely human, including morally relevant capacities. Among them, we find long-term planning, autonomous and flexible behavior, and the capacity to recognize the adequacy of an action, i.e., its relevance to achieving a specific goal. The existence of this type of AI may produce psychological uncertainty and even uneasiness that while not necessarily calling for an ontological redefinition of human identity and human nature as suggested by the posthuman philosophy (Braidotti 2013), has the potential to impact human self-perception, including moral self-perception.

## Conclusion

An ethical analysis of AI should include a reflection on both theoretical and practical issues. A prerequisite for addressing either is conceptual clarification of relevant key concepts. One of those critical concepts is intelligence, as is the similarities/differences between natural and artificial intelligence. A preliminary analysis of intelligence suggests that, to date, AI fails to exhibit several ethically relevant and critical features, i.e., having interests, capacity for preferences, and the ability to behave autonomously. This, in turn, suggests that certain claims about AI threats seem exaggerated or at least premature, even if the increasing use of AI in different societal domains raises legitimate, ethical questions.

Notwithstanding the impressive improvement of AI in recent years, notably the development of self-learning AI, human intelligence has several qualities that AI does not have. Time will tell whether this limitation is intrinsic to AI or only a matter of further development of the technology. In the meantime, it is reasonable to call for caution, and for the avoidance of both Neo-Luddism (i.e., a renewed form of opposition to technology) and of naïve enthusiasm about AI's possibilities.

## Footnotes

1 Stuart Russell and Peter Norvig suggest that historically two categories of AI definitions have been elaborated: human-centric and rationalist. In the first case AI is defined in comparison with humans, i.e. acting like a human or thinking like a human; in the second case AI is defined as characterized by an essential rationality or goal-directed behavior, i.e. thinking rationally or acting rationally. See (Russell and Norvig 2010).

2 The fact that emotions are key for a good relationship of assistance or education holds even if not all humans actually exhibit them in the best way. We are not arguing that AI fails to be emotionally involved while all humans are, but that to date AI lacks the ability to be emotionally involved while all humans at least potentially have such ability.

3 For a different view see (Danaher 2019).
